# Bidirectional Interactions Between Cervicovaginal Microbiota and Human Papillomavirus Drive Persistence and Disease Progression

**DOI:** 10.3390/ijms27125616

**Published:** 2026-06-22

**Authors:** Daniel Osmar Suárez-Rico, Lourdes del Carmen Rizo de la Torre, Martin Zermeño-Ruiz, Luis Ricardo Balleza-Alejandri, Jesús Jonathan García-Galindo, Héctor Montoya-Fuentes, Alberto Beltrán-Ramírez

**Affiliations:** 1 Departamento de Fisiología, Centro Universitario de Ciencias de la Salud (CUCS), Universidad de Guadalajara, Calle Sierra Mojada 950, Independencia Oriente, Guadalajara 44340, Mexico; daniel.suarez@academicos.udg.mx; 2 Laboratorio de Microbiología Molecular II, División de Medicina Molecular, Centro de Investigación Biomédica de Occidente, Instituto Mexicano del Seguro Social, Guadalajara 44100, Mexico; lourdes.rdlt@gmail.com; 3 Instituto de Terapéutica Experimental y Clínica (INTEC), Centro Universitario de Ciencias de la Salud, Universidad de Guadalajara, Guadalajara 44340, Mexico; luis.balleza3286@alumnos.udg.mx (L.R.B.-A.); jonathan.garcia@academicos.udg.mx (J.J.G.-G.); 4 División de Genética, Centro de Investigación Biomédica de Occidente, Instituto Mexicano del Seguro Social, Guadalajara 44340, Mexico; 5 Departamento de Farmacobiología, Centro Universitario de Ciencias Exactas e Ingenierías (CUCEI), Universidad de Guadalajara, Guadalajara 44430, Mexico; martin.zermeno@academicos.udg.mx

**Keywords:** human papillomavirus, cervicovaginal microbiota, vaginal dysbiosis, community state types, mucosal immunity, immune checkpoints, immunometabolism, lactate metabolism

## Abstract

Persistent high-risk human papillomavirus infection is a critical prerequisite for cervical intraepithelial neoplasia and cervical cancer, yet viral factors alone do not fully explain why most infections clear while a subset persists and progresses. Emerging longitudinal, multi-omics, and mechanistic evidence supports a plausible model in which the cervicovaginal microbiota is not a passive bystander but a functional determinant of mucosal immunity, epithelial barrier integrity, and local metabolic tone. *Lactobacillus*-dominant community states, particularly those enriched in *Lactobacillus crispatus*, are generally associated with lower pH, regulated inflammatory signaling, stronger barrier function, and a higher likelihood of HPV clearance. In contrast, anaerobe-enriched dysbiosis is linked to elevated pro-inflammatory cytokines, altered antigen presentation, immune checkpoint signatures consistent with T-cell dysfunction, and metabolic shifts involving lactate depletion and accumulation of short-chain fatty acids and other metabolites that can influence epithelial and immune-cell programs. Importantly, the interaction is bidirectional: hrHPV can remodel the microenvironment by suppressing host defense peptides and perturbing mucosal barriers, thereby reducing *Lactobacillus* fitness and reinforcing dysbiosis in a feed-forward loop that favors persistence and oncogenic progression. This review integrates functional ecology, longitudinal clinical evidence, immunological and metabolic mechanisms, and translational implications, highlighting opportunities for microbiome-informed risk stratification and adjunctive interventions, as well as key gaps requiring standardized longitudinal multi-omics and rigorously designed clinical trials.

## 1. Introduction

Persistent infection with high-risk human papillomavirus (hrHPV) represents the central biological event driving the development of cervical intraepithelial neoplasia (CIN) and invasive cervical cancer. While HPV acquisition is common and often transient, only a minority of infections progress to clinically significant disease. This discrepancy underscores the importance of host and microenvironmental determinants that modulate viral persistence beyond viral genotype and integration events alone [1]. Increasing evidence indicates that the cervicovaginal microenvironment—particularly the composition and functional state of the local microbiota—plays a critical role in shaping infection outcomes.

Longitudinal and meta-analytic studies consistently support that the composition of cervicovaginal microbiota is strongly associated with HPV persistence, lesion regression, and progression to CIN2+ and cancer. *Lactobacillus*-dominated communities, especially those characterized by *Lactobacillus crispatus*, are associated with enhanced viral clearance and regression of high-grade lesions [2,3]. In contrast, microbiota enriched in diverse anaerobic taxa—including *Gardnerella*, *Atopobium*/*Fannyhessea*, *Prevotella*, *Sneathia*, and *Fusobacterium*—are repeatedly linked to persistent hrHPV infection, progression to CIN2+, and even post-treatment recurrence [4,5]. A network meta-analysis of community state types (CSTs) reported that non–*Lactobacillus*-dominant or *L. iners*-dominant microbiota confer a two- to five-fold increased odds of hrHPV infection and cervical dysplasia compared with *L. crispatus*-dominant states [6]. These findings indicate that cervicovaginal microbial ecology constitutes a biologically relevant cofactor in the natural history of HPV infection.

Mechanistically, vaginal dysbiosis is associated with a chronic inflammatory milieu that qualitatively differs from effective antiviral immune responses. Elevated levels of pro-inflammatory cytokines—including IL-1α, IL-1β, IL-6, IL-8, TNF-α, and IL-15—have been consistently observed in *Lactobacillus*-depleted microbiota and HPV-positive women with dysplasia [7,8]. Functional studies indicate that dysbiosis-associated bacteria stimulate exaggerated TH17-skewed responses characterized by increased IL-17 production, which correlates with impaired viral clearance and disease progression [9,10]. Concomitantly, soluble immune checkpoint molecules such as PD-L1, TIM-3, and LAG-3 cluster with dysbiosis and cervical neoplasia severity, suggesting that chronic inflammation is accompanied by local T-cell dysfunction and immunoregulatory exhaustion [7]. These immune alterations overlap with HPV-mediated suppression of antiviral pathways and may synergistically promote sustained viral persistence.

Beyond immune modulation, emerging metabolomic evidence indicates that cervicovaginal dysbiosis is linked to alterations in metabolic pathways associated with oncogenic progression. Metabolomic profiling across the HPV–CIN–cancer continuum has revealed perturbations in amino acid, nucleotide, lipid, and ketone body pathways, including enrichment of sphingolipids and 3-hydroxybutyrate in dysplasia and cancer [11,12]. Such metabolic signatures correlate with inflammatory mediators and microbial diversity, indicating that microbiota-driven changes contribute to the immunometabolic reprogramming of the cervical microenvironment. Thus, the shift from a low-diversity, *Lactobacillus*-dominant ecosystem to a high-diversity anaerobic community extends beyond compositional change and involves functional reorganization of local metabolic and immune circuitry.

Importantly, the interaction between HPV and the cervicovaginal microbiota appears to be bidirectional. HPV infection has been shown to downregulate host antimicrobial peptides through the inhibition of NF-κB and Wnt/β-catenin pathways, resulting in decreased defensin expression and impaired innate immune recruitment [13]. Because these antimicrobial peptides serve as metabolic substrates for Lactobacilli, their reduction may compromise *Lactobacillus* survival and promote dysbiosis. This feed-forward loop—HPV-induced suppression of innate peptides, loss of *Lactobacillus* dominance, enhanced inflammation, and sustained viral persistence—provides a mechanistic framework linking viral oncogenesis with ecological disruption [5,13].

Collectively, current evidence supports a model in which the cervicovaginal microbiota functions as a dynamic regulator of HPV persistence and oncogenic progression through interconnected immune and metabolic pathways. A comprehensive molecular understanding of these interactions is essential to elucidate disease mechanisms and inform microbiome-targeted preventive and therapeutic strategies.

## 2. Functional Ecology of Cervicovaginal Microbiota

The cervicovaginal microbiota is a dynamic ecosystem whose structural organization and functional output shape mucosal barrier integrity and susceptibility to sexually transmitted viral infections, including human papillomavirus (HPV). Compared with other mucosal sites, this niche often exhibits relatively low bacterial diversity and frequent dominance of *Lactobacillus* species under eubiotic conditions [14,15]. Community profiling—initially enabled by 16S rRNA gene sequencing—led to the Community State Type framework, which captures recurrent microbial configurations that are increasingly recognized as functionally distinct ecological states rather than purely taxonomic labels [14,16]. This distinction matters because CSTs differ in their metabolic outputs (acidification versus SCFA/amine production), immune-modulatory effects, and capacity to preserve epithelial and mucus barrier functions—features that plausibly condition HPV acquisition and persistence [16,17].

### 2.1. Community State Types and Functional Meaning

**Figure 1:**
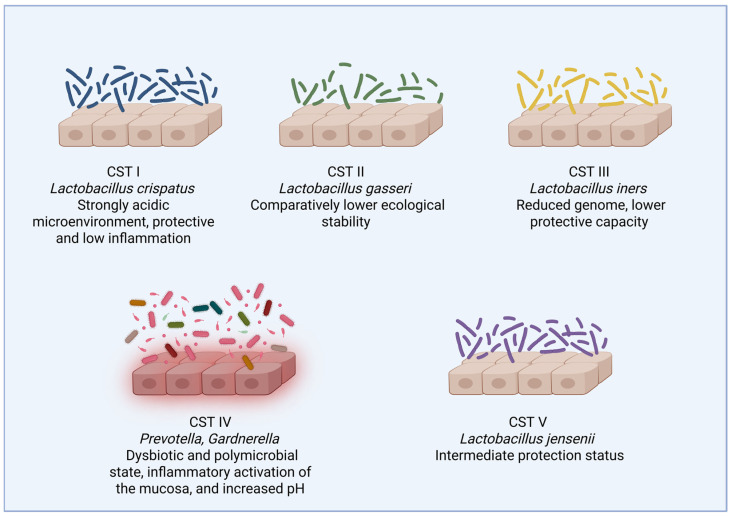
Functional overview of cervicovaginal community state types (CSTs). *Lactobacillus*-dominant CSTs (I, II, III, and V) differ in ecological stability and protective capacity, whereas CST IV reflects a high-diversity, anaerobe-enriched dysbiotic state associated with higher pH and inflammatory activation of the mucosa. CST I (*L. crispatus*) typically represents the most stable and protective configuration, whereas CST III (*L. iners*) frequently behaves as a transition-prone state. Created in BioRender. Beltrán-Ramírez, A. (2026) https://BioRender.com/o108bi6.

The conventional CST classification includes five major categories. CST I is typically dominated by *Lactobacillus crispatus* and is associated with low alpha diversity, robust lactic acid production (including a substantial D-lactate contribution), and a strongly acidic microenvironment (often pH < 4.5), features repeatedly linked to lower baseline inflammatory tone and reduced risk of persistent hrHPV detection and cervical dysplasia [2,16]. CST II is commonly dominated by *Lactobacillus gasseri* and generally shares protective features with CST I, although multiple datasets suggest that its ecological stability may be less robust than that observed with *L. crispatus*-dominant communities [14,15]. CST III is dominated by *Lactobacillus iners* and often represents a distinct ecological state: despite lactic acid production, its reduced genome, limited biosynthetic capacity, and frequent coexistence with anaerobes have been associated with increased transition propensity toward dysbiosis and less consistent protection [15,18]. CST IV is characterized by *Lactobacillus* depletion and higher diversity with strict or facultative anerobes (e.g., *Gardnerella*, *Atopobium*/*Fannyhessea*, *Prevotella*, *Sneathia*, and *Mobiluncus*), increased vaginal pH, and mucosal inflammatory activation—features repeatedly associated with higher susceptibility to STI acquisition and persistent HPV detection in longitudinal and mechanistic studies [2,3]. CST V is often dominated by *Lactobacillus jensenii* and is generally considered intermediate-to-protective, with lactic acid production and antimicrobial activity, although its stability and clinical associations appear more context-dependent than CST I [14,15] (Figure 1). Overall, CSTs should be interpreted as functional states with distinct immunometabolic profiles that modulate cervical epithelial homeostasis rather than static, purely compositional categories [14,16].

Importantly, CST IV should not be interpreted as a single homogeneous dysbiotic entity. Several subconfigurations exist within CST IV, differing in dominant anaerobic taxa, inflammatory profiles, metabolic output, and potential clinical associations. Therefore, CST IV-related risk should be interpreted in a context-dependent manner rather than as a uniform biological state. Similarly, *Lactobacillus iners*-dominant CST III should not be equated with frank dysbiosis. Rather, it represents a Lactobacillus-dominated but ecologically less stable and more transition-prone state than *Lactobacillus crispatus*-dominant CST I, with less consistent protective associations across studies.

### 2.2. Lactobacillus Dominance Versus Dysbiotic States

*Lactobacillus* dominance has been consistently linked to a protective cervicovaginal environment through synergistic metabolic, structural, and immunological mechanisms. Sustained acidification via lactic acid maintains a low pH that limits pathogen viability and shapes community stability. In addition, lactobacilli compete for epithelial adhesion sites and contribute to competitive exclusion through mechanisms that include lactic acid production, maintenance of low pH, bacteriocin-like activity, and modulation of epithelial innate responses. Although some vaginal lactobacilli can produce hydrogen peroxide under microaerophilic experimental conditions, its antimicrobial relevance in vivo is likely limited by the largely anaerobic and protein-rich cervicovaginal environment; therefore, H_2_O_2_ should be interpreted as a context-dependent rather than dominant protective mechanism [14,19]. Beyond direct antagonism, *Lactobacillus*-associated metabolites can modulate epithelial innate responses. In cervicovaginal epithelial models, lactic acid elicits immune responses distinct from those induced by dysbiosis-associated SCFAs, with lactic acid tending to suppress pro-inflammatory signaling relative to SCFA-driven profiles [20]. Importantly, lactic acid also enhances epithelial barrier integrity by promoting tight junction protein expression, supporting the concept that *Lactobacillus* dominance is functionally coupled to barrier preservation [21]. Organ-on-a-chip systems further reinforce these functional contrasts: *L. crispatus* consortia maintain low pH, preserve epithelial viability, and suppress inflammatory responses, whereas *Gardnerella*-rich consortia increase pH, injure the epithelium, and promote inflammation [22].

Conversely, CST IV dysbiosis is typically marked by anaerobe expansion and increased production of biogenic amines and mucinolytic enzymes, which raise pH (>4.5), degrade mucus components, and promote subclinical inflammation. This inflammatory state commonly involves cytokines such as IL-1β, IL-6, and TNF-α and is accompanied by barrier weakening, increasing basal cell exposure, and potentially facilitating HPV access to susceptible epithelial layers [17,19,23]. The ecological role of *L. iners* deserves particular scrutiny: although it defines a *Lactobacillus*-dominated CST, its functional profile differs from that of *L. crispatus*. *L. iners* predominantly produces L-lactate, lacks D-lactate dehydrogenase, depends on exogenous L-cysteine, and encodes aerolysin, all of which may contribute to epithelial irritation or inflammatory signaling in certain contexts [18,24]. Longitudinal observations repeatedly associate *L. iners* dominance with ecological instability and transitions toward dysbiosis, which may help explain its less consistent protective association against HPV persistence [3].

### 2.3. Ecological Stability and Resilience

The cervicovaginal microbiota exists in a dynamic equilibrium shaped by the host hormonal milieu, nutrient availability, mucosal immunity, and external perturbations. Ecological stability reflects the capacity to maintain structure under disturbance, while resilience reflects the ability to return to baseline after disruption—concepts that are particularly relevant given the frequent CST transitions observed in many individuals [14,15]. Estrogen-driven glycogen availability is a major stabilizing factor: estrogen promotes glycogen accumulation in vaginal epithelial cells, and after desquamation, glycogen is converted into fermentable sugars that support *Lactobacillus* growth and lactic acid production [14]. Accordingly, pregnancy and combined oral contraceptive use often correlate with increased *Lactobacillus* dominance and rapid stabilization [25]. By contrast, postpartum estrogen decline is associated with abrupt ecosystem perturbation and delayed recovery, often with prolonged high-diversity states [26], and menopause similarly tends to shift communities toward reduced *Lactobacillus* dominance [27].

Exogenous factors—including sexual activity, antibiotic exposure, intravaginal practices (e.g., douching), smoking, and other behavioral determinants—can disrupt equilibrium and precipitate dysbiotic transitions [14,28]. Clinically, baseline richness and biofilm-forming anaerobic consortia predict recurrence after standard BV therapy, emphasizing that dysbiosis can represent a resilient, difficult-to-displace ecological state rather than a transient imbalance [29,30]. Notably, metronidazole can reduce genital inflammation primarily by decreasing BV-associated bacteria rather than directly increasing *Lactobacillus* abundance, underscoring that durable recovery likely requires both removal of inflammatory anerobes and re-establishment of functionally protective *Lactobacillus* states [24,30]. Strain-level variation adds another layer: even within “*Lactobacillus*-dominant” states, functional gene content differs across strains and may influence adherence, metabolic competence, and transition propensity [16].

### 2.4. Metabolic Production and Biochemical Protection Mechanisms

Protection mediated by *Lactobacillus*-dominant ecosystems is fundamentally metabolic. Lactic acid is produced through the fermentation of glycogen-derived carbohydrates and exists as two optical isomers, D()-lactate and L(+)-lactate, with species-specific patterns: *L. crispatus* and *L. jensenii* typically contribute D- and L-lactate, whereas *L. iners* largely produces L-lactate [15,16]. The resulting low pH not only suppresses bacterial pathogens but may also influence viral particle stability and receptor interactions in a pH-dependent manner, a concept that remains plausible yet incompletely resolved for non-enveloped viruses such as HPV [31,32]. In parallel, lactic acid exerts immunomodulatory effects and enhances epithelial barrier integrity through tight junction upregulation [20,21]. Additional antimicrobial contributions—hydrogen peroxide production under microaerophilic conditions and bacteriocin synthesis—may reinforce competitive exclusion, although the magnitude of H_2_O_2_ activity in vivo is likely constrained by local anaerobiosis, suggesting that its protective value may be context-dependent [14,19].

### 2.5. Functional Integration with HPV Pathogenesis

Collectively, the functional ecology of the cervicovaginal microbiota appears to modulate multiple stages of HPV natural history, from acquisition to persistence and progression. Dysbiosis is associated with increased pH, heightened local inflammation, mucus and epithelial barrier compromise, and greater epithelial permeability—conditions that may facilitate viral access to basal cells and increase the likelihood of persistent infection [19]. During persistence, chronic low-grade inflammation may interfere with effective antigen presentation and local T-cell polarization, compromising immune clearance [19,23]. Over time, sustained inflammation and oxidative stress could plausibly contribute to genomic instability and favor oncogenic steps such as viral integration and epigenetic remodeling, although the causal chain and directionality remain areas where stronger mechanistic evidence is still needed [3,32]. In this sense, CSTs should be viewed as functional configurations with direct consequences for mucosal immunosurveillance and the probability of HPV clearance versus persistence, reinforcing the rationale for microbiome-informed risk stratification and targeted modulation strategies in HPV-associated disease.

Taken together, the functional ecology of the cervicovaginal microbiota establishes a dynamic immunometabolic framework in which community composition is inseparable from community function. The distinction between Lactobacillus-dominant and dysbiotic states is not merely taxonomic but reflects fundamentally different outputs in terms of mucosal acidification, epithelial barrier competence, innate immune tone, and metabolic substrate availability—features that collectively condition the cervicovaginal niche toward either viral clearance or sustained persistence. Critically, this ecological framework is not unidirectional: while microbiota composition shapes the conditions under which HPV is acquired and cleared, accumulating evidence indicates that HPV infection can in turn actively remodel microbiota composition through mechanisms addressed in subsequent sections. Understanding the ecological and functional determinants of community stability is therefore essential not only for interpreting the longitudinal clinical evidence reviewed in Section 3, but also for identifying the mechanistic nodes through which targeted microbiome modulation could interrupt the feed-forward loop linking dysbiosis, immune dysfunction, and oncogenic progression.

## 3. Longitudinal Clinical Evidence Linking Microbiota to HPV Persistence

The progression from transient HPV infection to persistent infection and, ultimately, to high-grade intraepithelial lesions is a multifactorial process involving viral, immunological, and microenvironmental determinants. Over the past decade, longitudinal studies have facilitated progress from cross-sectional associations to more robust temporal inferences about the role of the cervicovaginal microbiota in the natural history of HPV. The extant evidence indicates that vaginal dysbiosis is not merely an epiphenomenon but an active modulator of viral persistence and neoplastic progression [33,34].

### 3.1. Prospective Studies and Temporal Directionality

Prospective cohorts with serial follow-up using 16S rRNA gene sequencing have shown that women with a microbiota dominated by *Lactobacillus crispatus* have higher rates of HPV clearance than those with CST IV or *Lactobacillus iners* dominance [35]. These findings suggest that microbial configuration may precede and influence the probability of viral persistence, although definitive causal inference remains limited by the complexity of host–virus–microbiota interactions and by the observational nature of most available cohorts. The findings indicate that women classified as CST I (*L. crispatus*) consistently exhibit enhanced ecological stability and a considerably higher probability of viral clearance within 12–24 months [23]. In contrast, CST III states, predominantly characterized by *L. iners*, exhibit increased community variability and a higher frequency of transition to CST IV. Conversely, the prolonged persistence of CST IV has been associated with a reduced rate of spontaneous clearance of high-risk HPV. In multivariate analyses adjusted for age, sexual behavior, and co-infections, persistent dysbiosis remained an independent factor associated with persistent oncogenic HPV infection. Although absolute causality cannot be definitively established due to the complexity of host–virus–microbiota interactions, the temporal consistency of the findings and their biological plausibility support the hypothesis of a direct modulatory effect of the microbial ecosystem on the natural history of infection [34].

### 3.2. Bacterial Diversity, Viral Load, and Clearance

Increased bacterial diversity, a hallmark of CST IV, has been consistently correlated with higher HPV viral load and lower probability of clearance. Interrelated biochemical and immunological mechanisms underlie this phenomenon. The reduction in lactic acid and the production of biogenic amines by anaerobic bacteria increase the vaginal pH above 4.5. This alteration in pH changes the stability of cervical mucin and facilitates the exposure of basal cells susceptible to infection. Concurrently, microorganisms such as *Gardnerella*, *Atopobium*, and *Sneathia* activate Toll-like receptors (TLR2/TLR4), thereby promoting the release of proinflammatory cytokines such as IL-1β, IL-6, IL-8, and TNF-α [36,37]. This, in turn, engenders low-grade chronic mucosal inflammation, which can increase epithelial proliferation, induce microlesions, and promote cell differentiation-dependent viral replication. This sustained inflammatory activation can also alter local immune surveillance through functional exhaustion or dysfunctional polarization of dendritic cells and CD8+ T lymphocytes, reducing the efficiency of infected cell elimination. Consequently, quantitative studies have demonstrated that women with a microbiota not dominated by lactobacilli exhibit significantly elevated viral loads of high-risk HPV and diminished logarithmic reduction in viral DNA during follow-up. Conversely, *L. crispatus* dominance is associated with a progressive decline in viral load and a higher rate of negativization. These findings imply that high bacterial diversity is not only a risk marker but also a functional environment that facilitates viral amplification and episomal persistence of the HPV genome [23,38].

### 3.3. Microbial Signatures and Progression to CIN2+

The progression from persistent HPV infection to grade 2 or 3 cervical intraepithelial neoplasia (CIN2/CIN3) and cervical cancer involves viral DNA integration, sustained overexpression of E6/E7 oncoproteins, and genomic instability. The cervicovaginal microenvironment is associated with these processes. In this context, longitudinal studies have identified microbial signatures associated with an increased risk of clinical progression, including the overrepresentation of *Sneathia* spp., *Fusobacterium* spp., *Prevotella* spp., and *Atopobium vaginae* in women with CIN2+, a significant reduction in *Lactobacillus crispatus*, and an increase in local inflammatory biomarkers in the presence of persistent CST IV [38,39].

A compelling body of evidence establishes that the following mechanisms may link these microbial configurations to carcinogenesis:

- Chronic inflammation and oxidative stress, with sustained production of proinflammatory cytokines and reactive oxygen species capable of inducing epithelial DNA damage and facilitating viral integration [40].
- Disruption of the epithelial barrier is mediated by mucinolytic enzymes and bacterial proteases that degrade extracellular matrix components and tight junction proteins, such as claudins and occludins [41].
- Epigenetic modulation through bacterial metabolites that influence DNA methylation and histone acetylation alters genes related to proliferation and apoptosis [42,43].
- Possible synergy with viral oncoproteins by enhancing cell proliferation and reducing genomic repair capacity favors the accumulation of somatic mutations [37].

In prospective studies, women with persistently dysbiotic microbiota are more likely to progress to CIN2+ during follow-up than those who maintain lactobacillary dominance. Although not all cases of dysbiosis progress to neoplasia, the consistency of these associations supports a model in which the cervicovaginal microbiota influences viral persistence, increased viral load in contexts of chronic inflammation, and neoplastic progression by promoting viral integration and genomic damage. This model positions the microbiota not only as a potential prognostic biomarker but also as a modifiable therapeutic target [2,36]. However, controlled clinical trials are still needed to establish definitive causality and validate interventions based on microbiome modulation with proven clinical impact.

### 3.4. Clinical Integration and Translational Relevance

Longitudinal evidence supports a model in which the cervicovaginal microbiota significantly influences three critical stages in the natural history of HPV: viral persistence, by altering the immunometabolic microenvironment that compromises local immune surveillance; increased viral load in contexts of sustained chronic inflammation; and neoplastic progression, by favoring conditions that promote viral DNA integration and cumulative genomic damage [3]. This conceptual framework positions the microbiota not only as a potential prognostic biomarker but also as a modifiable therapeutic target. In this sense, interventions aimed at restoring states dominated by *Lactobacillus crispatus* could, in theory, increase viral clearance rates and reduce progression to high-grade intraepithelial lesions [37]. However, well-designed controlled clinical trials are still needed to establish a definitive causal relationship and validate strategies based on microbiome modulation with proven clinical impact.

### 3.5. Mixed HPV Infections and Microbial Dysregulation

Mixed infections comprising multiple high-risk human papillomavirus (hrHPV) genotypes introduce a profound layer of biological complexity that significantly impacts both the cervicovaginal microbiota architecture and the trajectory of disease progression. Emerging evidence indicates that coinfection with multiple hrHPV types correlates with expanded bacterial alpha-diversity, diminished *Lactobacillus* predominance, elevated pro-inflammatory cytokine profiles, and protracted viral persistence when juxtaposed with single-genotype infections [44]. This phenomenon likely reflects the additive or synergistic immunomodulatory effects exerted by multiple viral oncoproteins, which facilitate epithelial immune evasion, the downregulation of host antimicrobial peptides, and localized immune exhaustion. Clinically, patients harboring multi-genotype infections frequently exhibit elevated viral loads and an escalated risk of progression to high-grade CIN2+, as well as higher rates of post-therapeutic recurrence [45]. Furthermore, dysbiotic CST IV microenvironments may exacerbate this pathogenic cascade by perpetuating chronic mucosal inflammation and compromising antigen presentation pathways [46]. Notwithstanding these observations, the intricate crosstalk between specific viral genotype permutations and microbiota composition remains incompletely elucidated. Future longitudinal investigations are imperative to ascertain whether distinct microbiome signatures can be leveraged to refine risk stratification paradigms in patients with multi-type HPV infections [47].

### 3.6. Contradictory Evidence and Population Heterogeneity

Across cohorts, associations between Lactobacillus depletion, anaerobe enrichment, and HPV persistence are not uniform, and reported effect sizes can vary with population context and host/environmental modifiers (e.g., region, ethnicity, sexual behavior, coinfections, hormonal milieu, and immunosuppression) [48,49]. Likewise, Lactobacillus iners–dominant CST III is best interpreted as a context-dependent and often transition-prone state rather than a consistently “high-risk” configuration, as its relationship with HPV endpoints differs across studies and settings [29].

A further driver of variability is the internal diversity of CST IV, which encompasses multiple subconfigurations (often described as CST IV-A and IV-B) that differ in dominant anaerobes and functional outputs (e.g., sialidase activity, mucin degradation, biogenic amine production, and metabolite profiles) [14,16]. These differences likely contribute to heterogeneous patterns in pH, barrier integrity, and cytokine signatures, so a single CST IV label should be treated as an umbrella descriptor rather than a uniform inflammatory phenotype [50]. Methodological variation (sampling compartment, 16S region versus shotgun metagenomics, bioinformatic pipelines, and CST calling) also limits cross-study comparability. For these reasons, statements linking dysbiosis to inflammation and HPV persistence are most appropriately framed as probabilistic and context-dependent, and future work would benefit from standardized sampling, CST subtyping, and harmonized persistence/clearance endpoints with stratification by key host and population-level modifiers [48,49].

## 4. Dysbiosis and Mucosal Inflammation

### 4.1. Cytokine Profile

In the healthy cervicovaginal niche, *Lactobacillus*-dominant communities typically sustain a low-pH, acidic environment that is widely viewed as a cornerstone of mucosal homeostasis [51,52]. When *Lactobacillus* abundance declines and anaerobic taxa expand—commonly including *Gardnerella vaginalis*, *Fannyhessea* (*Atopobium*), and *Sneathia*—the ecosystem frequently shifts toward dysbiosis. This dysbiotic state is repeatedly associated with chronic inflammation, progressive immune dysfunction, and higher HPV prevalence or persistence across clinical and synthesis-level evidence [53].

A key conceptual advance came from a multi-omic study by Anahtar et al., which showed that cervicovaginal community structure is a major determinant of genital inflammatory profiles, supporting the idea that microbial ecology can set baseline cytokine tone in the female genital tract [54]. Subsequent clinical studies have further reported that anaerobe-enriched microbiota correlate with increased levels of pro-inflammatory cytokines—particularly IL-1β and TNF-α—whereas *Lactobacillus* (including *L. crispatus*) tends to show inverse associations with these mediators [55].

Among the cytokines commonly measured, IL-1β is often framed as a prototypical mucosal “alarmin” because it is closely linked to innate sensing and inflammasome-related pathways. At the cervicovaginal interface, elevated IL-1β has been repeatedly observed in dysbiotic and BV-like states, consistent with an epithelium–microbiota interface characterized by heightened danger signaling and recruitment of innate effector cells [17]. TNF-α similarly reflects innate immune activation and can amplify epithelial and stromal inflammatory programs, thereby sustaining a low-grade but persistent inflammatory milieu. In cohort studies examining microbiota–cytokine relationships, TNF-α increases alongside anaerobe-associated microbiota and shows inverse relationships with *Lactobacillus* dominance [56,57]. IL-6 remains mechanistically relevant given its bridge-like role between innate activation and downstream adaptive skewing, even when it is not consistently captured across all cervicovaginal cytokine panels. Notably, as disease progresses, local cytokine patterns may intensify or broaden: in cervicovaginal washing fluid from patients with cervical neoplasia, IL-1β and TNF-α (among other mediators) were significantly higher in cervical cancer compared with controls and CIN, underscoring that the local immune environment is measurably altered in established malignancy [58].

For a review centered on HPV persistence and oncogenic progression, these inflammatory “alarmins” are best interpreted not as uniformly protective but as components of a non-resolving inflammatory program that can coexist with ineffective antiviral clearance. Importantly, Shannon et al. reported that HPV infection and/or clearance was not associated with broad differences in cervical cytokines in their cohort, highlighting heterogeneity across populations and the influence of timing, sampling compartment, and host factors on the detectability of cytokine shifts. Nevertheless, the same study observed that HPV positivity aligned with high-diversity cervicovaginal microbiomes, consistent with broader observations that dysbiosis and genital inflammation often co-occur [59]. Taken together, the most defensible synthesis is that anaerobe-enriched dysbiosis frequently tracks with elevated IL-1β and TNF-α (and plausibly IL-6-dependent programs), and that this inflammatory background can contribute to immune dysregulation—suboptimal priming, T-cell dysfunction, and checkpoint upregulation—thereby favoring HPV persistence rather than efficient clearance [54].

### 4.2. Th1/Th2/Treg Polarization: From Non-Resolving Inflammation to Permissive Tolerance

A dysbiotic cervicovaginal environment characterized by persistent innate activation can coexist with counterregulatory programs that blunt effective antiviral effector function. Across HPV-driven cervical pathobiology, progression is commonly associated with the enrichment of regulatory T cells (Tregs) and an immunosuppressive cytokine milieu—particularly IL-10 and TGF-β—that can dampen antigen-specific cytotoxicity and promote local tolerance over clearance [1,60]. Reports spanning the spectrum from persistent infection to neoplasia describe Treg-associated phenotypes and IL-10/TGF-β signatures in cervical compartments, supporting the plausibility of a transition from early, potentially protective inflammation toward a more tolerogenic microenvironment during persistent infection and lesion evolution [23,60].

This polarization axis is often described as a shift away from Th1-like antiviral immunity toward Th2/Treg-skewed regulation, which is repeatedly emphasized as a hallmark of HPV immune escape and cervical carcinogenesis [23,60]. Mechanistically, chronic antigen exposure combined with ongoing innate stimulation at mucosal surfaces can promote T-cell dysfunction and preferential engagement of regulatory pathways that may limit tissue damage but inadvertently permit viral persistence [61,62].

### 4.3. Dendritic Cells and Antigen Presentation: Impaired Priming and Tolerogenic Instruction

HPV has evolved multiple strategies that reduce immunogenicity in infected epithelium, and viral proteins can interfere with innate pathways that would otherwise support effective activation and cross-priming [61,62]. Under these conditions, dendritic cells may receive incomplete maturation cues, resulting in suboptimal co-stimulation and weaker priming of cytotoxic T lymphocytes—an immunologic bottleneck plausibly favoring persistence [61,62]. As lesions develop, the antigen-presenting compartment may further adopt tolerogenic features reinforced by local regulatory cytokines such as TGF-β. In this context, TGF-β is often positioned as a key tissue signal shaping mucosal T-cell residency and functional programming in the genital tract, with implications for sustained local tolerance [63].

### 4.4. Immune Checkpoints (PD-L1, TIM-3, and LAG-3): Consolidation of Exhaustion and Viral Persistence

In HPV-related cervical neoplasia, PD-1/PD-L1 signaling is widely recognized as a central checkpoint axis associated with immune escape, and PD-L1 assessment is integrated into clinical frameworks guiding immunotherapy considerations in cervical cancer [64]. Pathology-based studies have evaluated PD-L1 alongside other inhibitory molecules across cervical squamous intraepithelial lesions and carcinomas, supporting the notion that checkpoint upregulation can appear along the continuum from premalignant disease to invasive cancer. Complementary observations indicate an increased prevalence of PD-1+ T cells within cervical lesions compared with peripheral blood, consistent with a locally constrained, exhaustion-prone phenotype that may undermine viral and tumor control [65].

A comprehensive mechanistic framework elucidates the nexus between dysbiosis-driven inflammation and immune checkpoint activation via a progressive cascade of localized immune dysregulation. The proliferation of anaerobic consortia upregulates pro-inflammatory cytokines, including IL-1β, IL-6, and TNF-α, whilst sustaining chronic Th17-polarized inflammation [66]. This milieu subsequently compromises dendritic cell maturation and attenuates antigen-presenting capabilities. The resultant suboptimal priming of cytotoxic T lymphocytes facilitates the focal expansion of regulatory T cells alongside the localized hypersecretion of IL-10 and TGF-β, thereby engendering a robustly tolerogenic microenvironment [67]. Such an immunosuppressive state catalyzes the upregulation of inhibitory checkpoint receptors—notably PD-L1, TIM-3, and LAG-3—culminating in the functional exhaustion of CD8+ T cells and the concomitant impairment of antiviral clearance. Concurrently, HPV oncoproteins E6 and E7 synergistically amplify these deleterious pathways by abrogating interferon signaling and further dismantling antigen-presentation machinery [68]. This dynamic orchestrates a pathogenic feed-forward loop that entrenches immune evasion, sustains viral chronicity, and accelerates neoplastic progression.

Considered collectively, the immune alterations associated with cervicovaginal dysbiosis and HPV persistence do not represent isolated or independent phenomena but rather a coordinated program of progressive immune subversion. The transition from innate activation and cytokine-driven inflammation, to T-helper polarization imbalance and impaired dendritic cell priming, to checkpoint-mediated T-cell exhaustion reflects a sequential erosion of the antiviral immune competence required for effective HPV clearance. Importantly, this immune dysfunction is not simply a downstream consequence of viral persistence; it is also actively shaped by the microbial ecology of the cervicovaginal niche, and the chronic inflammatory milieu sustained by dysbiosis can itself amplify and entrench these immunoregulatory programs.

## 5. Metabolic Reprogramming of the Cervical Microenvironment

### 5.1. Lactate as an Immunologic Modulator Beyond Acidification

A *Lactobacillus*-dominant cervicovaginal ecosystem is typically defined by high lactate output and low pH, whereas dysbiotic states enriched in anaerobes often show reduced lactate alongside increased alternative microbial metabolites, such as short-chain fatty acids (SCFAs) and biogenic amines. These metabolic shifts are not merely descriptive. BV-like states have been associated with higher concentrations of SCFAs and amines, whereas *Lactobacillus* dominance tends to align with lactate-rich profiles that support mucosal homeostasis [69,70].

From an immunologic perspective, lactate is increasingly treated as a context-dependent signaling metabolite capable of reshaping immune-cell differentiation and function. Contemporary syntheses in immunology and oncology emphasize that lactate can influence antigen-presenting and effector compartments, supporting either effective control or functional restraint depending on exposure dynamics and microenvironmental constraints [71,72]. In the cervicovaginal niche, lactate is reframed from a “protective byproduct” to a plausible immunometabolic rheostat: a *Lactobacillus*-rich, lactate-dominant milieu may help maintain barrier-compatible immune set-points, whereas loss of lactate dominance—together with accumulation of dysbiosis-associated metabolites—may contribute to inflammatory and regulatory programs that facilitate HPV persistence [69,71].

### 5.2. Bacterial Metabolites and HIF-1α/mTOR/Glycolysis: Convergent Metabolic Wiring

Cervicovaginal metabolites interface with canonical cellular metabolic circuits, including HIF-1α programs, PI3K/AKT/mTOR signaling, and glycolytic switching, which shape both epithelial biology and immune-cell effector capacity [73]. Mechanistic work links HPV oncoproteins with HIF-1α stabilization and/or target-gene expression, as well as the activation of growth and metabolic signaling consistent with a Warburg-like shift [70,71,72]. In addition, HPV E6/E7 have been implicated in the regulation of aerobic glycolysis through pathways affecting MYC-related programs [73,74]. Notably, much of the mechanistic evidence supporting HIF-1α stabilization, mTOR pathway engagement, and Warburg-like rewiring derives from cervical cancer tissues and cell-line models; therefore, extrapolation to the pre-neoplastic cervicovaginal niche should be made cautiously and framed as mechanistic plausibility rather than direct in vivo proof [48].

From the microbiota perspective, dysbiosis is associated with distinct exometabolite environments (SCFAs, amines, and organic acids) that can plausibly influence epithelial stress responses and local immune-cell metabolism. These microbial metabolite exposures may intersect with HIF-1α- and mTOR-linked pathways already primed by HPV oncogene activity, potentially reinforcing metabolic rewiring in the microenvironment [69].

### 5.3. Metabolomic Signatures in High-Grade Lesions: Toward Stage-Linked Molecular Phenotypes

Integrative profiling supports the feasibility of lesion-grade-associated metabolic stratification. A study combining vasginal microbiota analysis with cervicovaginal metabolomics reported that metabolic profiles reflect both cervical cancer status and microbiota context, and that specific features—including pathways linked to oxidative stress—can help distinguish normal cervix, CIN3, and invasive carcinoma [49]. Similarly, an analysis integrating metabolomics with 16S profiling in cervicovaginal lavage fluid from women with high-risk HPV infection identified multiple metabolite differences across negative, low-grade, and high-grade squamous intraepithelial lesion groups [75]. Tissue-based metabolomics provides orthogonal support, showing pathway-level alterations in cervical cancer specimens and indicating that metabolic remodeling is not limited to secretions but reflects tissue-level biology as well [76].

### 5.4. Tumor Metabolism and Dysbiosis: A Bidirectional, Reinforcing Loop

Tumor-associated metabolic remodeling can plausibly reshape—and be reshaped by—the cervicovaginal ecosystem. Cervicovaginal profiling indicates that metabolic signatures are jointly influenced by disease state and microbial community context, arguing against strictly one-directional models [49]. Emerging work links microbiome and metabolomic changes with clinically relevant HPV endpoints, suggesting that the metabolic landscape may track host–virus dynamics and could be modifiable in clinical contexts [77]. Consistent with this view, clinically oriented reviews increasingly position cervicovaginal microbiota composition, local metabolites, and HPV-related carcinogenesis as interdependent components of a shared microenvironmental system rather than isolated variables [78].

The metabolic landscape of the cervicovaginal microenvironment thus constitutes a third axis—alongside microbial ecology and immune regulation—through which dysbiosis and HPV oncoprotein activity jointly shape disease trajectory. The shift from a lactate-enriched, Lactobacillus-dominant metabolic profile toward one defined by SCFA accumulation, biogenic amine production, and HIF-1α- and mTOR-linked reprogramming is not merely a biochemical correlate of dysbiosis but a functionally relevant alteration that may contribute to epithelial stress responses, immune-cell effector capacity, and the microenvironmental conditions governing viral persistence and genomic instability. Crucially, this metabolic remodeling is itself subject to bidirectional influence: HPV oncoproteins can directly drive glycolytic reprogramming and alter the metabolic substrate availability of the cervicovaginal niche, while microbial metabolites can in turn amplify or attenuate oncoprotein-driven metabolic programs.

## 6. Bidirectional Interactions Between HPV and Cervicovaginal Microbiota

Accumulating evidence indicates that HPV infection can actively remodel the cervicovaginal ecosystem rather than merely coexist with it. Persistent infection—particularly with high-risk genotypes—has been repeatedly associated with reduced Lactobacillus dominance and a parallel expansion of bacterial vaginosis (BV)-associated anaerobes (e.g., *Gardnerella*, *Prevotella*, and *Sneathia*), often accompanied by increased microbial diversity [47,79]. Multi-study syntheses and mega-analytic approaches further support the concept that HPV-related disease states align with reproducible shifts toward anaerobe-enriched communities, consistent with an infection–microenvironment coupling that may influence persistence and progression [47,80]. Mechanistically, this remodeling has been linked to HPV-driven modulation of host defense peptides (HDPs), disruption of epithelial and mucus barrier integrity, and local immune reprogramming mediated by viral oncoproteins [78].

### 6.1. Modulation of Host Defense Peptides

Host defense peptides (HDPs) in the cervicovaginal mucosa constitute a dynamic barrier layer that becomes substantially altered during persistent HPV infection, particularly in the context of high-risk genotypes [79]. Human β-defensins (hBD1, hBD2, and hBD4) and α-defensins (HD-5 and HD-6) show markedly reduced expression in HPV-infected tissues compared with normal epithelium. In contrast, hBD3 appears to follow a distinct regulatory pattern, plausibly reflecting dual regulation via p53 and NF-κB signaling, which may account for its differential behavior relative to other defensins. In addition to defensins, other antimicrobial factors—including secretory leukocyte protease inhibitor (SLPI), S100A7, and elafin—have also been reported as downregulated, weakening the biological barrier against opportunistic pathogens [52,78].

A critical functional implication is that these peptides should not be viewed solely as broad-spectrum antimicrobials. Experimental work suggests that cervicovaginal lactobacilli (including *L. crispatus* and *L. jensenii*) can tolerate selected host defense peptides and, under defined in vitro conditions, may hydrolyze and internalize peptide fragments as nutrient sources [78]. Accordingly, it has been hypothesized that HPV-associated reductions in mucosal HDPs provide a biologically plausible route by which Lactobacillus ecological fitness could be diminished, potentially favoring shifts toward anaerobe-enriched communities. It is imperative to emphasize, however, that this feed-forward mechanism remains a hypothesis demonstrated primarily in vitro; its clinical relevance requires definitive in vivo confirmation in cervicovaginal secretions and robust longitudinal human studies before it can be considered an established fact [13].

Altered HDP expression has several biologically relevant consequences. First, reduced HDP levels can compromise mucus-associated antiviral trapping and decrease mucus viscosity, thereby facilitating HPV access to basal keratinocytes [78,81]. Second, HDP depletion may contribute to a dysbiotic inflammatory environment characterized by oxidative stress and increased pro-inflammatory cytokine secretion (including IL-6 and IL-8), which has been implicated in the progression of premalignant lesions [52,79]. Third, decreases in peptides such as elafin and S100A7 have been discussed as candidate non-invasive biomarkers associated with HPV persistence or dysplasia severity [79].

### 6.2. Disruption of the Epithelial and Mucus Barrier

The transition from *Lactobacillus*-dominant eubiosis to anaerobe-enriched polymicrobial dysbiosis—whether induced or facilitated by HPV—can compromise mucosal barrier integrity at multiple levels [78,82]. Dysbiotic taxa, particularly *Gardnerella*, *Prevotella*, and *Mobiluncus*, can produce sialidase (SNA), which hydrolyzes terminal sialic acid residues and disrupts mucus structure. Under physiological conditions, *Lactobacillus*-derived lactic acid is associated with increased cervical mucus viscosity and viral entrapment; loss of lactobacilli together with sialidase-mediated mucin degradation undermines this trapping barrier and facilitates virion access to basal keratinocytes [78,81]. Sialidase activity can also contribute to the degradation of additional defense factors, including secretory IgA, lactoferrin, and SLPI, further weakening mucosal protection [78,81].

Beyond mucus changes, anaerobe expansion and HPV-associated inflammation can alter epithelial architecture. *L. crispatus* preserves epithelial monolayer integrity, whereas dysbiotic states correlate with increased secretion of soluble E-cadherin, a biomarker consistent with epithelial barrier disruption [78]. In parallel, taxa such as *Fusobacterium nucleatum* and *Fannyhessea vaginae* have been implicated in activating innate immune pathways that destabilize tight junctions, thereby creating permissive niches for processes linked to persistence and oncogenic progression [52]. Dysbiosis is also associated with increased biogenic amines and reactive oxygen species (ROS), promoting chronic oxidative stress that can damage membrane proteins and foster genomic instability. This environment may increase the likelihood of HPV DNA integration into the host genome, a key event in carcinogenesis [80].

### 6.3. E6/E7-Mediated Local Immune Reprogramming and Feed-Forward Dysbiosis

Persistence of high-risk HPV depends on coordinated immune evasion and microenvironmental reprogramming. Viral oncoproteins E6 and E7 have been positioned as central drivers that shift the cervicovaginal niche away from protective eubiosis toward a pro-tumorigenic state characterized by HDP depletion, inflammatory signaling, and ecological conditions favoring anaerobic dominance [47]. A mechanistic anchor for this model is the observation that E7 can reshape the mucosal HDP landscape by interfering with NF-κB signaling: E7 interacts with NF-κB essential modulator (NEMO), promoting its proteasomal degradation, impairing p65 nuclear translocation, and suppressing inducible defensin expression (e.g., hBD2–4 and HD-5/6) in response to pro-inflammatory stimuli [13]. High-risk E7 can also interact—via its C-terminal domain—with CK1α and CK1γ isoforms, promoting their degradation. Loss of CK1 activity inhibits β-catenin phosphorylation at Ser45, leading to β-catenin stabilization and nuclear translocation, which enhances transcriptional programs (including c-MYC) that can repress constitutive HDPs, such as elafin and S100A7. In HPV-infected tissues, these peptides may be profoundly reduced relative to the normal epithelium [52].

**Figure 2:**
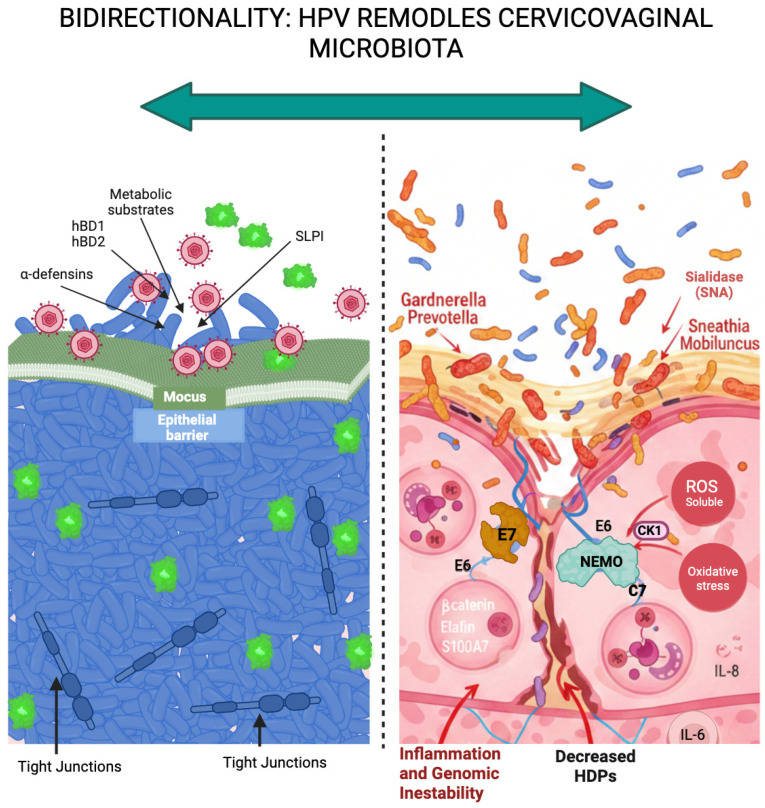
Bidirectional crosstalk between HPV infection and the cervicovaginal microbiota. In eubiosis, Lactobacillus-dominant communities sustain mucus and epithelial barrier integrity through lactate-dependent acidification, antimicrobial activity, and maintenance of host defense peptides, supporting tight junction function and immune homeostasis. During dysbiosis, expansion of anaerobic taxa (e.g., *Gardnerella*, *Prevotella*, *Sneathia*, and *Mobiluncus*) and increased sialidase activity compromise the mucus barrier, increase pH, and promote inflammatory activation and oxidative stress. High-risk HPV further remodels the microenvironment by downregulating HDPs via E6/E7-mediated interference with innate signaling pathways (including NF-κB/NEMO-related mechanisms), thereby reducing Lactobacillus fitness and reinforcing a feed-forward loop of dysbiosis, inflammation, and genomic instability that favors viral persistence and oncogenic progression. Created in BioRender. Beltrán-Ramírez, A. (2026) https://BioRender.com/5v5m8ac.

Functionally, HDP depletion can contribute to *Lactobacillus* decline and facilitate transition toward CST IV-like states dominated by anaerobes such as *Gardnerella* and *Sneathia*. This is particularly relevant because vaginal *lactobacilli* are auxotrophic for multiple amino acids and rely on proteolytic systems to hydrolyze and metabolize HDPs as substrates; therefore, reduced HDP availability can compromise *Lactobacillus* ecological fitness [78]. The combined loss of HDPs and *Lactobacillus*-mediated barrier protection can consolidate a permissive microenvironment characterized by chronic inflammation and oxidative stress, promoting host DNA damage, enhancing conditions that favor viral genome integration, and reducing dendritic cell and monocyte chemotaxis—thereby weakening innate immune surveillance and supporting viral escape [47,52]. In aggregate, these data support a feed-forward framework in which HPV-driven suppression of innate peptides reduces *Lactobacillus fitness*, promotes dysbiosis, and amplifies the inflammatory and barrier-disrupted niche that facilitates persistence and oncogenic progression (Figure 2) [78,80,81].

### 6.4. Host Genetic Background and Susceptibility to Microbiota-Mediated HPV Persistence

The complex interplay between HPV infection and the cervicovaginal microbiota is intrinsically modulated by the host’s genetic architecture, which dictates both microbial community dynamics and localized immunologic responses. Specifically, single nucleotide polymorphisms (SNPs) and allelic variations within genes governing antigen presentation (HLA class I and II), innate immune surveillance (TLR2, TLR4, TLR9), cytokine network regulation (IL-6, IL-10, TNF-α), and the secretion of epithelial defense peptides (β-defensins, SLPI, elafin) are significantly correlated with differential susceptibility to protracted HPV persistence and subsequent cervical neoplasia [83,84]. Such host genetic determinants likely govern the mucosal capacity to maintain Lactobacillus-predominant eubiosis, titrate inflammatory thresholds, and preserve the structural integrity of the epithelial barrier. For instance, genetic variations in Toll-like receptor (TLR) signaling pathways can distort the recognition of dysbiosis-associated anaerobic taxa, thereby exacerbating chronic inflammatory cascades. Concurrently, polymorphisms affecting defensin expression may perturb both intrinsic antimicrobial efficacy and the ecological fitness of *Lactobacillus* species [85]. Consequently, the multi-omic integration of host genomic data with cervicovaginal microbiome profiling holds substantial promise for refining precision risk stratification paradigms, thereby facilitating the identification of patient cohorts most likely to derive therapeutic benefit from microbiome-targeted interventions [86].

## 7. Therapeutic and Preventive Implications

Before considering translational applications, it is important to distinguish established clinical tools from emerging biomarkers and investigational microbiome-informed strategies. At present, cervicovaginal microbiome profiles, cytokine panels, and metabolomic signatures are not validated or standardized for routine clinical HPV screening, patient triage, surveillance, or treatment selection. Their current utility remains confined to investigative frameworks and the characterization of the microenvironmental state. However, therapeutic decision-making and risk stratification in HPV infection are conceptually moving away from a strictly virus-centric framework toward integrated laboratory models. In this perspective framework, predictive, preventive, and personalized (3P) medicine relies on combining emerging microenvironmental descriptors with validated cellular and molecular biomarkers to identify clinically meaningful patient subgroups and optimize future long-term surveillance.

Therapeutic decision-making and risk stratification in HPV infection are increasingly moving away from a strictly virus-centric framework toward integrated models that combine viral load and genotyping with cellular and molecular biomarkers and, increasingly, descriptors of the cervicovaginal microenvironment (microbiota composition, cytokines, and mucosal immunity) [87]. In this integrated view, 3P medicine relies on combined panels to identify clinically meaningful subgroups with different trajectories of persistence and progression and to guide targeted interventions that may include *Lactobacillus* restoration strategies and surveillance using circulating free HPV DNA (cfHPV-DNA) in oncologic settings [83].

### 7.1. Combined Biomarkers: Microbiome Signature, Cytokines, and HPV Viral Load

Current HPV risk assessment relies primarily on extended genotyping, quantitative viral load, and tissue markers (e.g., p16/Ki67, MCM, methylation), with cfHPV-DNA serving as an emerging oncological monitoring tool [84,88]. Evidence regarding vaginal microbiota and cytokine profiling suggests potential incremental value for stratification and monitoring, although these components remain largely investigational in most clinical pathways [89].

Metagenomic and epidemiologic data consistently indicate that *Lactobacillus*-dominant cervicovaginal states—particularly *L. crispatus*-dominant CST I—are associated with a lower prevalence of high-risk HPV (hrHPV), a higher probability of clearance, and reduced risk of progression to CIN2+ [90]. Conversely, anaerobe-enriched dysbiosis (CST IV) promotes a pro-inflammatory microenvironment that favors HPV persistence and high-grade lesions [91]. Longitudinal evidence suggests a temporal link, as ecological transitions toward CST IV often precede or accompany HPV detection and CIN progression [5].

Multi-omic profiling reveals that HPV-associated dysbiosis aligns with elevated pro-inflammatory cytokines (e.g., IL-1β, IL-6, TNF-α) and epithelial injury, whereas *L. crispatus*-dominant states remain lactate-enriched and immunologically regulated. [92,93]. This inflammatory pattern correlates with higher HPV viral loads and increased epithelial expression of E6/E7 oncoproteins, reinforcing the concept that an “inflammatory–dysbiotic” state can act as a determinant of persistence [90,91].

From a virologic standpoint, multiple cohort studies indicate that combining genotyping with viral load improves predictive performance beyond HPV positivity alone. High viral loads—particularly of HPV-16—track with increased risk of CIN2+ and persistence, while sharp post-treatment viral load declines correlate with response and lower recurrence [94,95]. In cervical cancer, detection and quantification of cfHPV-DNA using liquid biopsy approaches (including ddPCR and other ultrasensitive platforms) have shown value for monitoring response to radiochemotherapy and for detecting minimal residual disease or early relapse [52,88].

Tissue biomarkers provide added biological specificity. Overexpression of p16INK4a and Ki67 reflects E7-driven deregulation of the pRb pathway and aberrant proliferation, and panels combining p16/Ki67 (dual stain), MCM, and host methylation markers such as FAM19A4/miR124-2 can improve discrimination between transient infections and lesions with true progressive potential [84]. In liquid-based cytology, combined assessment of MCM and viral expression markers such as E4 has been proposed to distinguish clinically insignificant lesions from truly progressive HSIL [96,97].

Conceptually, integrating microbiome signatures (CST classification, pathobiont detection) and local cytokines with established virologic, tissue (e.g., p16/Ki67), and systemic (cfHPV-DNA) biomarkers provides a plausible framework for future multiparametric panels. Although no such integrated algorithm is currently validated for routine care, incorporating CST data and a Lactobacillus dominance index could theoretically refine progression prediction for intermediate-risk genotypes or borderline cytology, potentially reducing unnecessary colposcopy referrals [91,93,98].

### 7.2. 3P Medicine (Predictive, Preventive, and Personalized) in HPV Infection

Applied to HPV, 3P medicine centers on (i) quantifying individualized progression risk, (ii) deploying preventive interventions tailored to the risk profile, and (iii) personalizing management of lesions and cancer based on combined molecular and microbial features.

In the predictive domain, contemporary risk models already integrate extended genotyping, viral load, cytology, dual-stain markers, methylation signatures, and clinical factors to estimate CIN2+/cancer probability and guide referral and follow-up decisions [84]. Adding microbiota parameters (CST, *Lactobacillus* dominance index, pathobiont signals) and local cytokine profiles could further refine prediction, particularly in young women with incident multi-type infections; patients with intermediate-risk genotypes and mild cytology; and immunosuppressed individuals or those with comorbidities affecting mucosal immunity [99].

In the preventive domain, personalization translates into optimizing prophylactic vaccination schedules (including adaptations for immunosuppressed individuals), tailoring screening strategies (intervals, test type, and self-sampling where appropriate), and implementing targeted vaginal/oral probiotics or, in the future, vaginal microbiota transplantation (VMT) in women with persistent dysbiosis and elevated HPV/cervical cancer risk [97,100].

In the personalized therapeutic domain, integrating virologic, cellular, and microbiome biomarkers could prospectively guide nuanced clinical decisions. This integration supports balancing conservative versus excisional management for HSIL to preserve fertility, tailoring radiochemotherapy intensity based on cfHPV-DNA clearance and immune context, and informing the biomarker-driven selection of immunotherapies, potentially combined with adjunctive microbiome modulators (e.g., precision probiotics, VMT) [83,101].

Although many of these applications remain prospective, the expanding availability of multi-omics and integrative analytic workflows makes it plausible that HPV management will increasingly adopt 3P schemes in which the cervicovaginal microbiome is considered alongside viral and molecular biomarkers [102].

### 7.3. Risk Stratification Based on Expanded Panels

Most CIN2+ risk stratification frameworks currently combine extended genotyping, viral load, Bethesda cytology, p16/Ki67 dual staining, and clinical factors such as age, persistence, immunosuppression, and smoking [99,103,104,105]. Incorporating microbiota and cytokine data could refine progression probabilities in clinical subgroups. For example, a “high biological risk” profile—combining persistent HPV-16/18, high viral load, abnormal tissue markers (≥ASC-H/HSIL), and a pro-inflammatory CST IV dysbiosis—would confer the highest CIN3/cancer probability, justifying immediate intervention [84].

Intermediate risk, modulable by microbiota: women with HPV-31/33/58 (“intermediate-risk” genotypes), ASC-US/LSIL, and moderate viral load, but with *L. crispatus* or *L. gasseri* dominance and low inflammatory cytokines—longitudinal data suggest many clear infections without significant progression, supporting conservative surveillance rather than invasive procedures [90,106].

Microbiota/cytokine-reclassified risk: individuals with formally “lower-risk” genotypes for CIN2+ (e.g., HPV-51/56/59/66) but a strongly dysbiotic CST IV-B microbiota and elevated cytokines may carry higher progression risk than predicted by genotype or cytology alone, potentially warranting more intensive follow-up [91,107].

Finally, combining cfHPV-DNA with cervicovaginal microbiota and cytokine parameters could, in the future, help discriminate patients requiring therapeutic intensification (e.g., adjuvant immunotherapy) from candidates for de-escalation strategies [83,88].

### 7.4. Clinical Trial Evidence and Translational Validation

Despite their conceptual promise, translating microbiome-informed interventions into clinical practice is limited by a lack of large-scale randomized controlled trials (RCTs). Currently, multiple ongoing trials are evaluating microbiota-targeted strategies—including probiotic supplementation (e.g., *L. crispatus* M247), vaginal microbiota transplantation (VMT), and therapeutic E6/E7 vaccines (e.g., VGX-3100, GX-188E)—to definitively assess their efficacy in promoting HPV clearance and lesion regression [108,109]. Currently, clinical guidelines do not incorporate cervicovaginal microbiota profiling. Future integration demands harmonized CST frameworks, validated biomarkers, and rigorous trials demonstrating reproducible clinical efficacy on endpoints such as CIN regression and recurrence prevention.

## 8. Knowledge Gaps and Future Directions

### 8.1. Lactobacillus Restoration: Evidence on Vaginal and Oral Probiotics

Restoration of *Lactobacillus*-dominant vaginal states is increasingly explored as an adjuvant strategy to promote HPV clearance and reduce progression risk. Controlled studies and clinical trials have examined diverse probiotic formulations with heterogeneous, yet overall encouraging results [110].

The clinical efficacy of probiotic interventions in HPV-positive women demonstrates a strong dependency on strain specificity, route of administration, treatment duration, baseline vaginal CST, and host-specific factors, including hormonal profile and sexual behavior. Crucially, species within the Lactobacillus genus do not exhibit uniform ecological or immunological mechanisms. For instance, *Lactobacillus crispatus* is predominantly correlated with enhanced ecological stability, robust D-lactate synthesis, and a more sustained restoration of vaginal eubiosis when contrasted with *Lactobacillus iners* or specific *Lactobacillus rhamnosus* formulations [111,112].

Mechanistically, vaginal administration facilitates direct recolonization of the cervicovaginal niche, whereas oral supplementation may induce indirect modulatory effects via the gut–vagina axis and systemic immune pathways. Furthermore, therapeutic duration emerges as a critical determinant: longitudinal studies utilizing vaginal *Lactobacillus rhamnosus* BMX 54 for six months or more report a significantly more consistent rate of HPV-DNA clearance compared to short-course regimens or brief oral therapies [113]. However, the lack of dosage standardization across the current literature introduces significant heterogeneity, thereby constraining direct empirical comparisons and meta-analytic syntheses. Ultimately, these findings indicate that probiotic efficacy must not be misconstrued as a broad class effect; rather, it constitutes a highly strain-specific and protocol-dependent intervention that necessitates rigorous methodological standardization prior to broad clinical endorsement.

The heterogeneity of probiotic interventions highlights the importance of strain specificity, route of administration, and treatment duration (Table 1).

Among vaginal approaches, prolonged application of *Lactobacillus rhamnosus* BMX 54 is one of the most extensively studied strategies. In women with bacterial vaginosis or vaginitis and concurrent HPV infection, standard antimicrobial therapy (metronidazole or fluconazole) followed by vaginal administration of *L. rhamnosus* BMX 54 for 3 versus 6 months suggested that longer regimens were associated with higher HPV-DNA negativization rates and more durable restoration of vaginal eubiosis [116]. Although these studies were not consistently randomized against placebo, they collectively imply that duration and route of administration may be critical determinants of sustained microenvironmental effects and, potentially, HPV natural history.

In the oral setting, a randomized, double-blind, placebo-controlled trial evaluated daily *L. rhamnosus* GR-1 and *L. reuteri* RC-14 in women with high-risk genital HPV infection and reported no statistically significant difference in HPV clearance between probiotic and placebo arms, while observing fewer mildly abnormal cytology results (ASC-US/LSIL) and fewer unsatisfactory smears after 6 months, suggesting improvement in inflammatory tone and cytology quality without a clear direct antiviral effect [117].

Formulations centered on *Lactobacillus crispatus* M247 have also shown promising signals in uncontrolled observational studies, including marked reductions in HPV positivity and transitions from CST IV/III toward CST I, consistent with effective recolonization and ecosystem restoration. However, limited sample sizes and lack of control groups warrant cautious interpretation, and multicenter randomized trials are underway to evaluate *L. crispatus* M247 using CST and 16S sequencing endpoints [114].

More recently, oral supplementation with *Lactiplantibacillus plantarum* Probio87 in HPV-positive women over 12 weeks increased vaginal *Lactobacillus* and reduced pathogens such as *Streptococcus* and *Candida*, with parallel improvements in symptomatology and psychological well-being. While primary endpoints were microbiome modulation (vaginal and intestinal), the findings reinforce the plausibility of a gut–vagina axis that could influence immune responses relevant to HPV [115].

Mechanistically, *Lactobacillus* and related beneficial strains may support HPV control through multiple, non-mutually exclusive pathways. They sustain low vaginal pH via L- and D-lactic acid production, potentially limiting pathogen replication and shaping viral and immune gene expression programs [93]. They also produce hydrogen peroxide and bacteriocins with activity against vaginosis-associated bacteria and certain viruses; moreover, *Bifidobacterium adolescentis* SPM1005-A has shown in vitro antiviral activity with reduced HPV-16 E6/E7 expression [118]. Additional small clinical observations suggest that probiotic administration may modulate cytokines such as TNF-α, IL-6, and IL-8 in specific contexts, supporting immunomodulatory potential, while broader immunologic syntheses highlight immunization-based prevention and treatment frameworks for papillomavirus-related cancers [119,120]. Overall, despite supportive signals, evidence for direct effects on HPV clearance and CIN regression remains limited and heterogeneous, and phase II/III trials that integrate genotyping, viral load, and microbiota/cytokine endpoints are required before probiotics can be recommended as standard anti-HPV therapy [110].

### 8.2. Immunotherapy and Vaccination: Potential Impact of the Microbiome

Prophylactic L1 virus-like particle vaccines (bivalent, quadrivalent, and nonavalent) remain highly effective for preventing incident HPV infections and related neoplasia, but they do not have therapeutic effects on established infections or pre-existing lesions [87,100]. Observational evidence indicates that vaccination after excisional treatment of high-grade lesions reduces recurrence risk, likely by preventing reinfection at the surgical bed [121]. Direct evidence that cervicovaginal microbiota composition modifies immune responses to intramuscular prophylactic HPV vaccination is currently lacking. Therefore, this review does not propose that vaginal lactobacilli directly regulate antigen presentation following prophylactic vaccination. Rather, the potential relevance of the cervicovaginal microbiota is more plausible in the context of established infection, therapeutic E6/E7-directed vaccination, checkpoint blockade, or lesion regression, where the local mucosal and tumor immune microenvironment may influence effector-cell recruitment, immune exhaustion, and treatment responsiveness. Accordingly, microbiome–immunotherapy combinations should be regarded as investigational and require prospective validation [93,98].

For therapeutic vaccination, multiple platforms targeting E6/E7 (including DNA and RNA vaccines, peptide/protein formulations, viral/bacterial vectors, and dendritic cell-based strategies) have shown immunogenicity and efficacy signals in premalignant lesions, but none has yet achieved regulatory approval [87,121,122]. Among the most advanced approaches, DNA vaccines, such as VGX-3100 and GX-188E, have demonstrated CIN2/3 regression and HPV clearance in subsets of patients, while also illustrating constraints imposed by immune evasion and local immunosuppressive microenvironments [123].

Preclinical work continues to illustrate the potential ceiling of next-generation immunotherapies under favorable conditions. In models using a non-integrating lentiviral vaccine (Lenti-HPV-07) encoding detoxified HPV-16/18 E6/E7, complete tumor eradication with a single intramuscular dose was achieved in animals bearing HPV-induced tumors, accompanied by robust infiltration of effector and tissue-resident CD8+ T cells, tumor microenvironment remodeling, long-term memory induction, and synergy with PD-1 blockade [124]. These results underscore that immunotherapy efficacy is critically dependent on the local immunologic context.

The cervicovaginal microbiota may shape this context via (i) microbial products (e.g., LPS, proteases, and sialidases) that promote the recruitment of myeloid-derived suppressor cells and regulatory T cells, (ii) barrier disruption and neoantigen exposure, and (iii) metabolic shifts (lactate and SCFAs) that influence T- and NK-cell function [92]. Although clinical studies directly associating cervicovaginal microbiota profiles with response to therapeutic vaccines or checkpoint inhibitors in HPV-associated cancers remain scarce, experience in other solid tumors supports a meaningful role for the microbiome—particularly the intestinal microbiome—as a determinant of anti–PD-1 efficacy [125,126]. In this context, evaluating whether restoration of cervicovaginal eubiosis can potentiate E6/E7 vaccination or PD-1/PD-L1 blockade in HPV-associated cervical and anal cancer represents a priority direction for translational research [123,124].

## 9. Key Scientific Gaps and Future Research Priorities

Despite considerable advancements in elucidating the crosstalk between the cervicovaginal microbiota and human papillomavirus (HPV) persistence, several critical knowledge gaps continue to impede clinical translation.

First, the standardization of CST classification remains inadequately defined. Inconsistencies across sequencing platforms, taxonomic resolution, sampling topography, and bioinformatics pipelines preclude direct cross-study comparability and compromise reproducibility. The establishment of harmonized frameworks for functional CSTs, coupled with strain-level genomic resolution, is a prerequisite for clinical operationalization [127].

Second, delineating causality remains a formidable challenge. The preponderance of current evidence is derived from cross-sectional analyses or inadequately powered longitudinal cohorts, confounding the distinction between dysbiosis as a primary driver of HPV chronicity versus secondary microbial destabilization induced by persistent infection. Adequately powered, prospective, multicenter longitudinal investigations are imperative to resolve temporal directionality [128].

Third, the application of integrated multi-omics frameworks remains nascent. Transitioning beyond purely compositional microbiota profiling necessitates the concurrent interrogation of metagenomic, metabolomic, transcriptomic, proteomic, and host immune landscapes to unearth robust mechanistic pathways and prognostic biomarkers [129].

Fourth, there is a distinct paucity of large-scale, randomized controlled trials (RCTs) evaluating microbiome-targeted therapeutics. Interventions such as precision probiotics, VMT, post-ablative microbiome restoration, and microbiome-modulated immunotherapies demand rigorous empirical validation utilizing standardized clinical endpoints—namely, HPV clearance, regression of CIN, mitigation of recurrence, and long-term survival metrics [130].

Fifth, the integration of host genomic architecture into prevailing pathogenic models remains deficient. Genetic polymorphisms dictating mucosal immunocompetence, epithelial barrier integrity, and inflammatory rheostat functions likely exert a profound influence on both microbiota resilience and therapeutic responsiveness [131].

Finally, contemporary clinical guidelines for cervical cancer screening and management have yet to assimilate microbiota-derived biomarkers, notwithstanding mounting evidence of their prognostic utility. Future algorithmic paradigms must coalesce CST profiling, inflammatory signatures, viral load quantification, and host molecular biomarkers to actualize precision medicine frameworks.

Addressing these strategic priorities is paramount for transcending descriptive correlative associations, ultimately paving the way for microbiome-informed prophylaxis, advanced risk stratification, and personalized therapeutic regimens in HPV-driven malignancies.

## 10. Search Strategy and Evidence Appraisal

This article is a narrative review guided by SANRA principles. The literature searches were performed in PubMed/MEDLINE, Scopus, and Web of Science on 15 March 2026 (last updated on 10 April 2026). The primary time window was January 2020 to December 2025; seminal pre-2020 references were retained selectively when they represented foundational contributions repeatedly cited in contemporary literature.

### 10.1. Search Strings and Filters

For each database, we used predefined Boolean queries combining four domains: (i) HPV infection and persistence, (ii) cervicovaginal microbiota ecology, (iii) mucosal immunity/inflammation, and (iv) metabolism/immunometabolism and interventions.

### 10.2. Record Handling and Selection Workflow

Records were exported and deduplicated in Zotero. Titles and abstracts were screened for relevance to the review’s central questions on bidirectional HPV–microbiota interactions, followed by full-text assessment of potentially eligible articles. As this is a narrative review, we did not apply formal inclusion/exclusion criteria by study design, sample size, or a predefined risk-of-bias instrument; instead, selection prioritized mechanistic relevance, methodological rigor, recency, and representativeness of the evidence base.

### 10.3. Evidence Appraisal and Use of AI-Enabled Tool

To address differences in evidentiary strength across the literature, we classified key studies supporting each major claim as randomized trials, longitudinal cohorts, cross-sectional studies, or mechanistic in vitro/animal work, and we explicitly avoid causal language when the evidence is observational. AI-enabled tools were used only for citation discovery (citation network expansion); all candidate records were manually retrieved from primary sources, and each final citation was verified by DOI, title, and abstract relevance before inclusion.

## 11. Conclusions

The pathogenesis of persistent high-risk HPV infection and subsequent cervical carcinogenesis involves complex interactions beyond viral factors alone. The evidence reviewed here supports a model in which the cervicovaginal microbiota acts as a biologically plausible, yet currently exploratory, cofactor associated with mucosal immunity, epithelial barrier integrity, and local metabolic homeostasis. Lactobacillus-dominant community states, particularly those enriched in *Lactobacillus crispatus*, consistently correlate with a protective microenvironment characterized by sustained acidification, regulated innate immune signaling, and an enhanced probability of viral clearance. Conversely, anaerobe-enriched dysbiotic states are associated with a permissive niche that may facilitate HPV persistence and oncogenic progression through chronic mucosal inflammation, altered antigen presentation, and dysbiosis-linked metabolic reprogramming.

Crucially, emerging in vitro and observational data suggest this relationship may be bidirectional. High-risk HPV infection might contribute to remodeling the cervicovaginal microenvironment through E6/E7-mediated suppression of host antimicrobial defense peptides and the subversion of mucosal immune responses. These virus-driven alterations theoretically deprive *Lactobacillus* species of key ecological supports, potentially facilitating a shift toward anaerobe-enriched communities. In turn, dysbiosis could reinforce inflammation and immune dysfunction, hypothetically consolidating a feed-forward loop in which viral persistence and ecological imbalance become mutually sustaining processes.

Several limitations of the current evidence base must be acknowledged. Most available studies remain cross-sectional or associative, limiting the ability to define the precise temporal sequence or absolute causality of microbiota–HPV interactions. Furthermore, the volume of evidence supporting microbiota-driven modulation of HPV outcomes is substantially greater than that characterizing HPV-driven remodeling of the microbiota, reflecting an important asymmetry in the current state of the field.

While the mechanistic framework synthesized in this review highlights the cervicovaginal microbiota as a potential future focus in the continuum of cervical neoplasia, realizing the translational value of precision probiotics, vaginal microbiota transplantation, or microbiome–immunotherapy combinations will require rigorously designed, adequately powered randomized controlled trials. Until robust clinical trial data confirm reproducible efficacy and safety, the concept of the cervicovaginal microbiota as an actionable therapeutic target must be treated as strictly exploratory and investigational.

## Figures and Tables

**Table 1:** Comparative summary of probiotic interventions for HPV persistence and cervicovaginal microbiota restoration.

Probiotic Strain/Formulation	Route of Administration	Duration	Main Reported Outcome	Proposed Mechanism	Main Limitation	Reference
*Lactobacillus* *rhamnosus* BMX 54	Vaginal	3–6 months	Higher HPV-DNA negativization, improved restoration of vaginal eubiosis, reduced recurrence of bacterial vaginosis/vaginitis	Direct recolonization of vaginal niche, lactic acid production, restoration of Lactobacillus-dominant CSTs, reduction in local inflammation	Limited placebo-controlled trials; heterogeneous study design	[113]
*Lactobacillus* *rhamnosus* GR-1 + *Lactobacillus reuteri* RC-14	Oral	6 months	No significant increase in HPV clearance; fewer mildly abnormal cytology results (ASC-US/LSIL), improved smear quality	Gut–vagina axis modulation, systemic immune regulation, indirect support of vaginal Lactobacillus restoration	Limited direct antiviral effect on HPV clearance	[112]
*Lactobacillus* *crispatus* M247	Vaginal/Oral (depending on study)	Variable (generally ≥ 3 months)	Reduction in HPV positivity, transition from CST III/IV to CST I, improved vaginal microbiota stability	Strong D- and L-lactate production, enhanced epithelial barrier integrity, stable recolonization of protective CST I	Mostly observational studies; lack of large randomized trials	[114]
*Lactiplantibacillus plantarum* Probio-87	Oral	12 weeks	Increased vaginal Lactobacillus abundance, reduced pathogens (*Streptococcus*, *Candida*), improved symptoms and quality of life	Gut–vagina axis modulation, indirect microbiome stabilization, immune regulation	HPV clearance not primary endpoint	[115]

## Data Availability

No new data were created or analyzed in this study. Data sharing is not applicable to this article.

## Abbreviations

The following abbreviations are used in this manuscript:

HPV	Human papillomavirus
hrHPV	High-risk human papillomavirus
CIN	Cervical intraepithelial neoplasia
HSIL	High-grade squamous intraepithelial lesion
LSIL	Low-grade squamous intraepithelial lesion
CST	Community state type
BV	Bacterial vaginosis
VMB	Vaginal microbiota
CVM	Cervicovaginal microbiota
SCFAs	Short-chain fatty acids
HDPs	Host defense peptides
hBD	Human beta-defensin
SLPI	Secretory leukocyte protease inhibitor
TLR	Toll-like receptor
DC	Dendritic cell
Treg	Regulatory T cells
Th	T helper cell
IFN	Interferon
IL	Interleukin
TNF	Tumor necrosis factor
PD-1	Programmed cell death protein 1
PD-L1	Programmed death-ligand 1
TIM-3	T-cell immunoglobulin and mucin-domain containing-3
LAG-3	Lymphocyte activation gene 3
HIF-1α	Hypoxia-inducible factor 1-alpha
PI3K	Phosphoinositide 3-kinase
AKT	Protein kinase B
mTOR	Mechanistic target of rapamycin
ROS	Reactive oxygen species
cfHPV-DNA	Circulating cell-free HPV DNA
ddPCR	The droplet digital polymerase chain reaction
VMT	Vaginal microbiota transplantation
STI	Sexually transmitted infection
LPS	Lipopolysaccharide
